# Data on cell growth inhibition induced by anti-VEGF siRNA delivered by Stealth liposomes incorporating G2 PAMAM-cholesterol versus Metafectene® as a function of exposure time and siRNA concentration

**DOI:** 10.1016/j.dib.2016.06.064

**Published:** 2016-07-06

**Authors:** Nasim Golkar, Soliman Mohammadi Samani, Ali Mohammad Tamaddon

**Affiliations:** a Pharmaceutics Department, School of Pharmacy, Shiraz University of Medical Sciences, Shiraz 71345, Iran; b Center for Nanotechnology in Drug Delivery, Shiraz University of Medical Sciences, Shiraz 71345, Iran; c Pharmaceutical Nanotechnology Department, School of Pharmacy, Shiraz University of Medical Sciences, Shiraz 71345, Iran

**Keywords:** Anti-VEGF siRNA, Cell growth inhibition, Polyamidoaminedendrimer, Liposome

## Abstract

In this data article, carboxyfluorescein-loaded liposomes were prepared and purified from free carboxyfluorescein using gel filtration chromatography in the first part. In the next part, following preparation of anti-VEGF siRNA loaded liposomes incorporating hydrophobically modified G2 PAMAM dendrimer (G2-Chol_40%_) (Golkar et al., 2016) [1], the cell growth inhibition induced by the formulations (siRNA/Metafectene complexes and siRNA loaded liposomes incorporating hydrophobic G2) was evaluated at two exposure times through MTT assay in a breast cancer cell (SKBR-3) and compared by two-way ANOVA.

**Specifications Table**

**Table:** 

Subject area	*Biology*
More specific subject area	*Gene delivery*
Type of data	*Graphs, figures*
How data was acquired	*Gel filtration chromatography, Fluorimetry (Fluorescence plate reader, Infinite 200, TECAN, Austria), UV Spectroscopy(UV-Visible ELISA plate reader, Biotek, USA)*
Data format	*Analyzed*
Experimental factors	*Free carboxyfluorescein was excluded from carboxyfluorescein loaded liposomes by gel filtration chromatography and fluorimetry. Cell growth inhibition was evaluated following transfection at two time exposures for two formulations by MTT assay using UV Spectroscopy.*
Experimental features	*There was no difference between two time exposures in transfection assay. Growth inhibition was siRNA concentration dependent.*
Data source location	School of Pharmacy, Shiraz University of Medical Sciences, Shiraz, Iran
Data accessibility	*Data are provided with this article*

**Value of the data**

• Our data shows possible growth inhibition effects of the anti-VEGF siRNA loaded liposomes incorporating hydrophobically modified G2 PAMAM dendrimer at different siRNA concentrations in a breast cancer cell line that is comparable to a commercial transfectant (Metafectene).
• The cell growth inhibition decrease dependently by increasing the siRNA concentration. However, there is no difference between 4 and 13 h time exposures of cells with formulations in a 72-h transfection assay. The data may valuable for researches concerning the effect of siRNA concentration and time exposure for evaluation of the biological effects such as growth inhibition.

## 1 Data

**Fig. 1:**
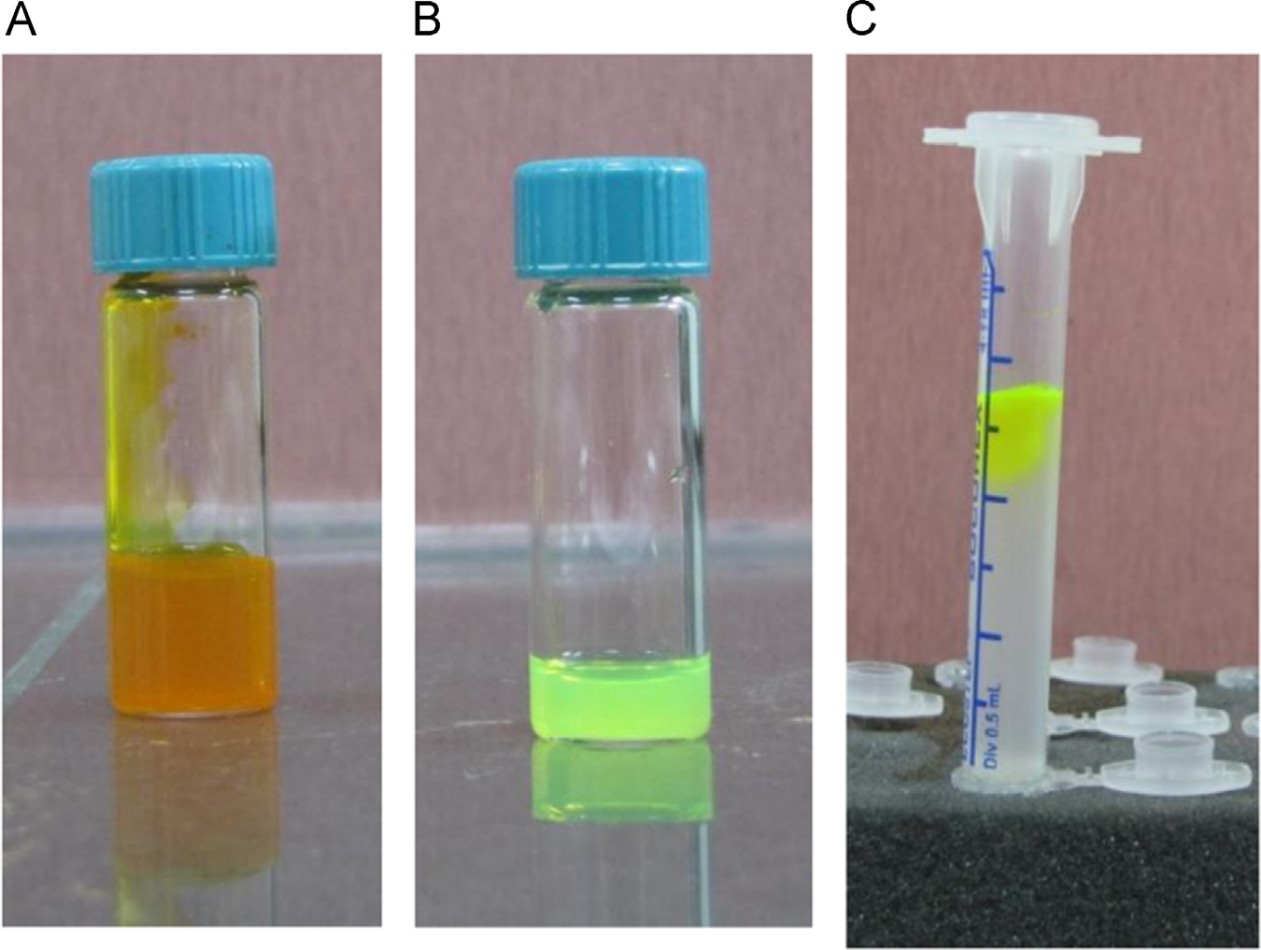
Carboxyfluorescein 38 mM in 25 mM HEPES buffer pH=7.4 (A), carboxy fluorescein encapsulated 5 mM liposomes in 25 mM HEPES buffer pH=7.4 (B), and gel filtration Sephadex G50 column (C).

**Fig. 2:**
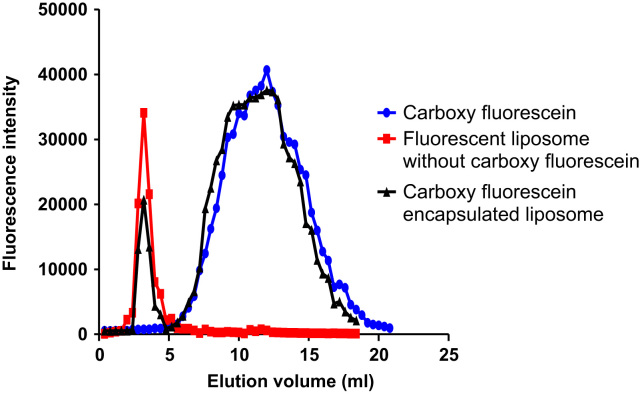
Gel filtration chromatograms of 38 mM free carboxy fluorescein, 5 mM rhodamine-labeled liposomes (1 mol% rhodamine-PE), and carboxyfluorescein encapsulated liposomes. Fractions of 7-11 and 13-50 (each fraction=400 µl) corresponds to the liposomes and free carboxyfluorescein eluted from the Sephadex G50 column (1 cm×5 cm, diameter×length) with 20 mM HEPES buffered saline solution (pH=7.4).

**Fig. 3:**
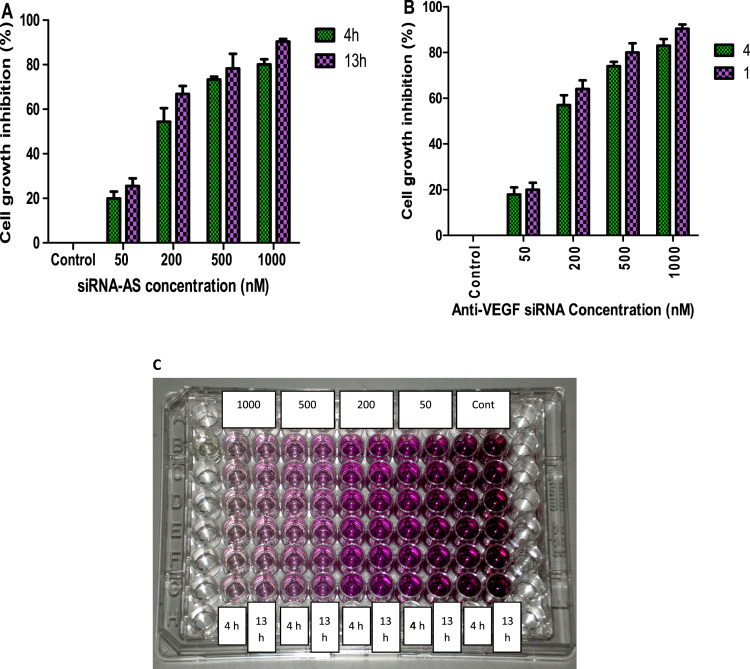
Growth inhibition following 4 or 13 h exposure of SKBR-3 cells with different concentrations of (A) anti-VEGF siRNA (siRNA-AS)/Metafectene complexes and (B) anti-VEGF siRNA (siRNA-AS) loaded liposomes incorporating G2-Chol_40%_. The cells were then incubated with fresh complete medium for 72 h at 37 °C before MTT assay. (C) MTT-based growth inhibition assay of SKBR-3 cells with various concentrations of anti-VEGF siRNA (siRNA-AS)/Metafectene complexes. Data represents mean±SD for triplicates.

Carboxyfluorescein-loaded liposomes were purified from free carboxyfluorescein using gel filtration chromatography (Figs. 1 and 2). Then, hydrophobically modified G2 PAMAM dendrimer was incorporated into the liposome and siRNA was loaded in the prepared nanocarrier. The cell growth inhibition following transfection with siRNA/Metafectene complexes and siRNA loaded liposomes incorporating hydrophobic G2 was evaluated at two exposure times (Fig. 3A and B, respectively) and compared by two-way ANOVA.

## 2 Experimental design, materials and methods

### 2.1 Material

1,2-Distearoyl-sn-glycero-3-phosphocholine, 1,2-distearoyl-sn-glycero-3-phosphoethanolamine-N-(methoxy(polyethylene glycol)-2000) and 1,2-dioleoyl-sn-glycero-3-phosphoethanolamine were purchased from Lipoid (Germany). G2 PAMAM dendrimer, Sephadex G50, carboxy fluorescein and 3-(4,5-dimethylthiazol-2-yl)−2,5-diphenyltetrazolium bromide (MTT) were from Sigma-Aldrich (USA). Metafectene® was from Biontex (Germany), anti-VEGF siRNA (siRNA-AS) was synthesized by Bioneer Co. (Korea) and SKBR-3 cell line was obtained from the Pasteur Institute (Iran).

### 2.2 Liposome preparation

Carboxyfluorescein-loaded liposomes were prepared by thin layer hydration method and extrusion [1], [2].

### 2.3 Gel filtration chromatography

Carboxy fluorescein-loaded liposomes were purified from free carboxyfluorescein using gel filtration chromatography (Fig. 1). The gel media consists of spherical porous particles of controlled pore size through which molecules separated based on their different molecular sizes. The stock suspension of Sephadex G50 (50 mg/ml) in HEPES buffered saline was incubated at 90 °C for 5 min and then washed several times. The beads were washed by adding, agitating and decanting the buffer, and then the suspension was poured into a column (1 cm×5 cm, internal diameter×length) and allowed to remain stationary for at least 24 h in order to pack the column for gel filtration chromatography. The column was equilibrated and eluted several times with 25 mM HEPES buffer (pH=7.4). To verify the suitability of the column properties for separation of the liposomes from free carboxy fluorescein, 400 µl of carboxy fluorescein solution (38 mM) and 0.5% rhodamine-labeled liposomes (5 mM) were individually loaded into the column. The elution fractions were collected successively and they were analyzed by a fluorescence plate reader at the excitation and emission *λ*_max_ of 492 nm and 517 nm for carboxy fluorescein and 560 nm and 583 nm for rhodamine. Accordingly, the carboxyfluorescein-loaded liposomes were purified from free carboxyfluorescein by collecting the liposome containing fractions (fractions of 7-11). Carboxy fluorescein-loaded liposomes were used for further investigation of the liposome leakage after incorporation of hydrophobically modified G2 into liposome structure [1].

As shown in Fig. 2, the liposome fraction was separated from free carboxyfluorescein by gel filtration chromatography, which was performed to remove unloaded carboxyfluorescein molecules. The free carboxyfluorescein was eluted from the column starting from 5.2 ml to 20 ml. Similar to the rhodamine-labeled liposomes, purified carboxyfluorescein loaded liposomes were eluted from the column from 2.8 ml after the first 2.4 ml (void volume) elution of the column which was continued to 4.4 ml. The dilution factor for the purified liposomes was equivalent to five.

### 2.4 Preparation of SiRNA loaded liposome incorporating G2-chol_40%_

Following incorporation of G2-Chol_40%_ into the liposome structure at a specific N(amine)/L(lipid)/P(phosphate), siRNA was loaded through ethanol drop method as previously published [1].

### 2.5 Cell growth inhibition assay

The cell growth inhibition was determined for siRNA/metafectene complexes and siRNA loaded liposomes incorporating G2-Chol_40%_ each at different siRNA concentrations of 50, 200, 500 and 1000 nm at similar N/P ratio of 2 after 4 h or overnight transfection (13 h) and successive 72-h post-incubation with fresh medium. SKBR-3 cells were seeded in 96-well plates (a density of 2×10^4^ cells/well). Following 24 h incubation at 37 °C, the cells were treated with each sample (1/10 dilution in serum-free culture media) at 37 °C for 4 h. The medium was changed with fresh complete culture medium and the cells were incubated for 72 h. Then, 100 µl of 0.5 mg/ml MTT solution was added to each sample. Following 3 h incubation at 37 °C, the light absorbance was read at *λ*=570 and 650 nm by a microplate reader. The percentage of growth inhibition was measured from Eq. (1):

(1) $$\mathrm{Growth}\;\mathrm{inhibition}\left(\%\right)=\left\{1-\left[\frac{A_{\mathrm{Sample}570}-A_{\mathrm{sample}650}}{A_{\mathrm{control}570}-A_{\mathrm{control}650}}\right]\right\}\times 100$$

where *A*_Sample__570_ and *A*_Sample650_ present to the absorbance of treated samples at the respective *λ* of 570 and 650 nm; *A*_Control570_ and *A*_Control650_ are the corresponding absorbance of untreated control cells.

As illustrated in Fig. 3, the anti-VEGF siRNA concentration showed a significant effect on the cell growth (*P*<0.0001). The cell growth decreased by increasing the concentration for both metafectene and liposomes. However, the difference between 4 h and 13 h transfection was not found significant (*P*>0.05).

### 2.6 Statistics

The data were expressed as mean±standard deviation. The results were at least in triplicate. Statistical analysis was performed by Graphpad Prism Software Inc. version 5.04. Data were compared by analysis of variance (ANOVA). The differences were considered statistically significant when *P*<0.05.
